# Time and frequency domain analysis of physiological features during autonomic dysreflexia after spinal cord injury

**DOI:** 10.3389/fnins.2023.1210815

**Published:** 2023-08-28

**Authors:** Ana Karina Kirby, Sidharth Pancholi, Zada Anderson, Caroline Chesler, Thomas H. Everett, Bradley S. Duerstock

**Affiliations:** ^1^Weldon School of Biomedical Engineering, Purdue University, West Lafayette, IN, United States; ^2^Krannert Cardiovascular Research Center, Division of Cardiovascular Medicine, School of Medicine, Indiana University, Indianapolis, IN, United States; ^3^School of Industrial Engineering, Purdue University, West Lafayette, IN, United States

**Keywords:** spinal cord injury, autonomic dysreflexia, sympathetic nerve activity, autonomic functions, heart rate variability

## Abstract

**Introduction:**

Autonomic dysreflexia (AD) affects about 70% of individuals with spinal cord injury (SCI) and can have severe consequences, including death if not promptly detected and managed. The current gold standard for AD detection involves continuous blood pressure monitoring, which can be inconvenient. Therefore, a non-invasive detection device would be valuable for rapid and continuous AD detection.

**Methods:**

Implanted rodent models were used to analyze autonomic dysreflexia after spinal cord injury. Skin nerve activity (SKNA) features were extracted from ECG signals recorded non-invasively, using ECG electrodes. At the same time, blood pressure and ECG data sampled was collected using an implanted telemetry device. Heart rate variability (HRV) features were extracted from these ECG signals. SKNA and HRV parameters were analyzed in both the time and frequency domain.

**Results:**

We found that SKNA features showed an increase approximately 18 seconds before the typical rise in systolic blood pressure, indicating the onset of AD in a rat model with upper thoracic SCI. Additionally, low-frequency components of SKNA in the frequency domain were dominant during AD, suggesting their potential inclusion in an AD detection system for improved accuracy.

**Discussion:**

Utilizing SKNA measurements could enable early alerts to individuals with SCI, allowing timely intervention and mitigation of the adverse effects of AD, thereby enhancing their overall well-being and safety.

## 1. Introduction

There are approximately 17,810 new SCI cases each year in the United States (National Spinal Cord Injury Statistical Center, 2016). SCI is not only devastating because of paralysis, but secondary complications also impede one’s overall well-being (Ahuja et al., 2017). One of the most severe secondary complications arising from autonomic nervous system dysfunction (ANS) in high-level SCI is AD, which is characterized by uncontrolled sympathetic nerve hyperactivity. Approximately 70% of individuals with an SCI at or above the T6 level experience AD, which is typically triggered by noxious stimulation below the level of injury (Bycroft et al., 2005). Common causes or triggers of AD include bladder dilation, bowel distention, and restrictive clothing (Murray et al., 2019). These triggers cause nerve impulses to be sent to the spinal cord, activating the sympathetic nervous system, constricting blood vessels, and increasing blood pressure. The increase in blood pressure is detected by baroreceptors, which send signals to the brain to oppose the rise in blood pressure by slowing the heart and opening blood vessels. This compensatory control is accomplished through the parasympathetic vagus nerve activation of the heart and the blood vessels above the level of injury. However, this parasympathetic response cannot overcome the constricted vessels below the level of injury, resulting in prolonged hypertension (Karlsson, 2006). If not treated promptly, AD can cause damage to major organs and can be fatal (Eldahan and Rabchevsky, 2018).

Being familiar with triggers and symptoms is necessary to identify AD, which can be problematic for newly injured patients. A survey of individuals with an SCI and their families revealed 41% had never heard of AD (McGillivray et al., 2009). Clinically, the gold standard for AD detection is an increase of 20 mmHg or more in systolic blood pressure (SBP), but constantly monitoring a patient’s blood pressure is burdensome and impedes daily activities (D. W. Popok et al., 2017). Currently, no noninvasive monitoring system is available to aid with the detection of AD. Therefore, AD is not typically monitored outside of the clinical setting. Current technological advances in noninvasive electronic sensors in the biomedical domain allow for the development of a wearable AD detection system for continuous monitoring. Noninvasive AD detection requires two components: an array of noninvasive physiological sensors and a machine learning model trained using participant data to determine the onset of an AD event.

An AD detection system was previously created for persons with SCI using a Microsoft Band™ smartwatch device, galvanic skin response (GSR), skin temperature, and heart rate. A machine learning model was trained using data from persons with SCI and was based on their presentation of symptoms, such as sweating, headaches, and cold and clammy skin. This system detected the onset of AD with 94.1% accuracy and a 4.9% false negative rate in participants performing regular daily activities (Suresh and Duerstock, 2018, 2020). A comprehensive evaluation of other noninvasive physiological features that may be superior to these smartwatch-based sensors was assessed, particularly through time domain analysis of SKNA. Temporal measures of sympathetic and parasympathetic activity including ISKNA (integrated skin nerve activity), number of bursts, percentage of the number of normal-to-normal values (medianNN), and root mean square of successive differences (RMSSD) were shown to optimally form machine learning models with high accuracy of AD detection (93.4%; Suresh et al., 2022a,b). In another study (Sagastibeltza et al., 2022), the occurrence of AD in patients with SCI was investigated using clinical and physiological data. The study successfully developed a classification system that could identify patients at risk of AD with an 80% accuracy rate, indicating the promising potential of these techniques for diagnosing AD in SCI patients.

In previous studies conducted on rats, the frequency domain of both SKNA and HR have been examined in the context of SCI. These investigations have revealed notable differences in the frequency patterns exhibited by these signals before and after SCI. Specifically, it has been observed that during episodes of AD, the very low frequency (VLF) and low frequency (LF) components of HRV are dominant, while the high frequency (HF) components are more prominent in the baseline state (Carnevali et al., 2013; Silva et al., 2017). In human and large animal models, the commonly used frequency ranges for LF and HF bands are 0.04–0.15 Hz and 0.15–0.40 Hz, respectively (Cívicos Sánchez et al., 2021). However, in the rat model, the frequency bands differ slightly. The specific frequency ranges used for LF and HF bands in the rat model are as follows: LF (0.2–0.75 Hz) and HF (0.75–2.5 Hz; Zajączkowski et al., 2014; Silva et al., 2017). Furthermore, research involving rats and bats has extensively explored a wide range of frequencies, spanning from 0 to 10 Hz. Interestingly, the findings reveal that both rat and bat species exhibit dominance exceeding 2 Hz (Morrison, 1999; Reynolds et al., 2019). This intriguing observation suggests the presence of distinct and significant frequency patterns in these animals, highlighting the relevance and importance of investigating frequency responses beyond the conventional HF range. These ranges have been established through research and studies conducted on rats.

The prompt detection of AD is of utmost importance. A quick and noninvasive AD detection system capable of providing advance warning, even if within a range of seconds to minutes, would be highly valuable in anticipating a rise in SBP. Such a system would allow individuals to take immediate action, such as raising the head of the bed and seeking assistance to address the AD trigger, thereby potentially mitigating the onset of hypertension.

Using a rat model of SCI, the timing of SKNA was compared to the increase in blood pressure, monitored in real-time through implanted telemetry and non-invasive ECG electrodes, establishing a correlation. Further characterization of AD events was investigated using frequency domain analysis of HRV and ISKNA. Previous frequency domain analyses have determined disease or ill health states compared to normal states based on the emergence of dominant frequencies (Physick-Sheard et al., 2000). In addition, selecting dominant frequency bands as potential features in creating machine-learning models could lead to more accurate AD detection (Suresh et al., 2022b).

One of the key contributions of this work is the estimation of the onset time of SKNA features in comparison to other HRV features when AD occurs. By examining the timing of SKNA activity alongside other HRV measures, a further understanding of the temporal relationships and potential associations between these physiological markers was gained. This analysis provides a deeper understanding of the interplay between SKNA and other HRV features. Secondly, the study focuses on analyzing the features in both the time domain and frequency domain, allowing for a comprehensive assessment of the significant differences between AD events and baseline conditions. By examining these domains, the research provides valuable insights into the distinct characteristics and patterns associated with AD and baseline state.

## 2. Methods

### 2.1. Animal model

Male Sprague Dawley rats (*n* = 9), purchased from Envigo (Indianapolis, IN), were used in this study. Before arriving at the research facility, four-month-old rats weighing 350–400 grams were implanted with a single-pressure biopotential implant (HD-S11 Implant, Data Sciences International, USA). The entire telemetry implantation surgery was conducted at Envigo, Indianapolis. Male rats were selected exclusively for the study due to the high incidence of SCI in men, who account for around 80% of all cases and tend to sustain injuries at an earlier age (Raguindin et al., 2021). All animals were individually housed in plexiglass cages with straw bedding and had access to food and water. The rats were acclimated to the experimental setup, which included electrodes, a restraining jacket, and a plastic holder with air holes (HLD-RL model, Kent Scientific, USA), over a four-week period using a previously published protocol (Kirby et al., 2023). Acclimation was necessary to measure ANS parameters in conscious animals without undue stress.

### 2.2. Experimental procedures

#### 2.2.1. SCI surgery and post-surgery care

A dorsal laminectomy was performed at the T3 level in each rat, exposing the spinal cord. Using specially made forceps, the spinal cord was compressed for 15 s to induce injury. The compression at the T4 level caused ischemia, replicating common clinical injuries as described by Blight (1991). Following the SCI, the rats experienced hindleg motor function loss, which was assessed through a toe pinch test, while retaining the ability to use their forelimbs.

Post-surgery, the rats’ bladders were manually expressed, and they received 15 mL of lactated Ringer’s solution subcutaneously. The rats were closely monitored and kept on a heating pad until fully awake and alert. They were then placed in cages with ALPHA-dri® bedding to prevent abdominal irritation caused by movement. Meloxicam and Baytril were administered once a day for 3 days post-surgery to prevent infection and alleviate pain. During the first week after surgery, the rats’ bladders were expressed three times a day to prevent urinary tract infections. Trained personnel monitored and weighed the rats daily. To encourage eating, Ensure® nutrition, peanut butter, and apple sauce were provided since weight loss is commonly observed after SCI. If the animals lost more than 20% of their initial body weight prior to the SCI surgery, they were euthanized. A 5-day recovery period was provided after surgery before commencing the experiments.

#### 2.2.2. CRD procedure

**Figure 1:**
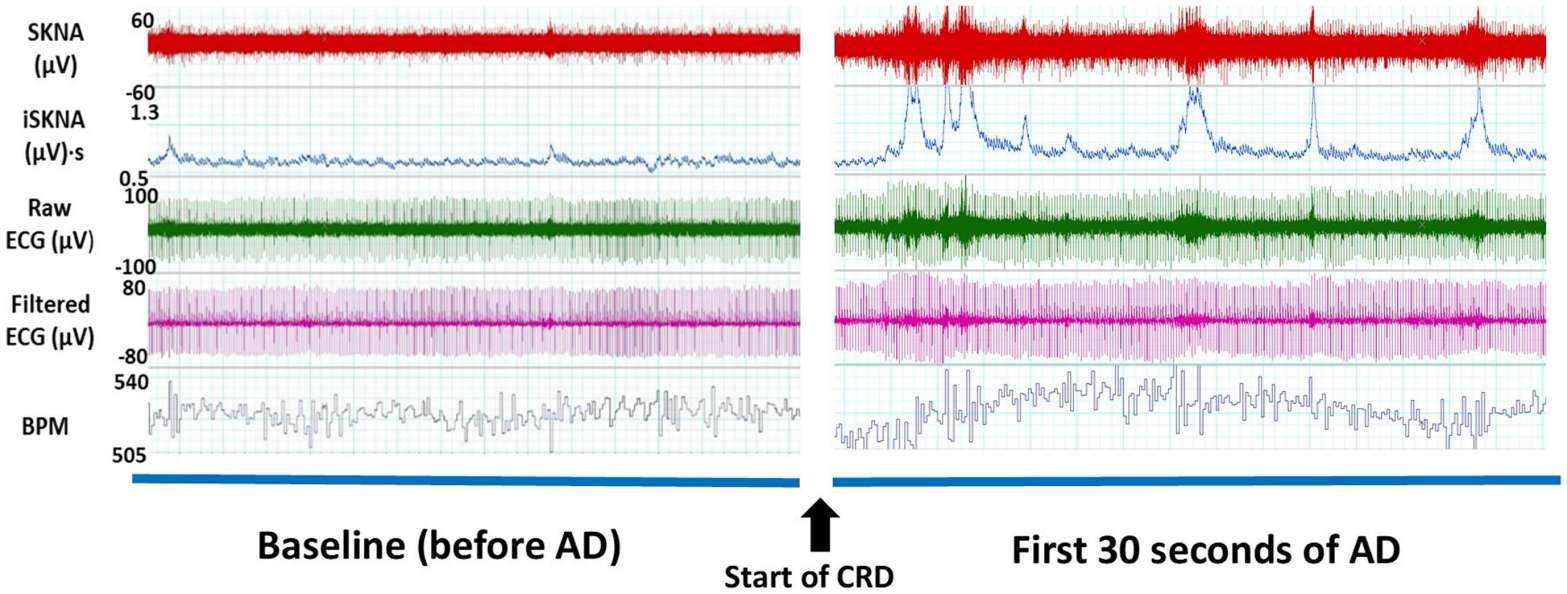
Thirty-second windows of electrocardiography (ECG) signals collected from a representative subject for one autonomic dysreflexia (AD) event from non-invasive electrodes. An increase in skin nerve activity (SKNA) and integrated skin nerve activity (ISKNA) is seen within the first 30 s of the onset of AD.

The experimental setup for the CRD protocol involved anesthetizing the rats with 4% isoflurane and inserting a lubricated balloon-inflated catheter 2 cm into their rectums (Rabchevsky et al., 2011; Sachdeva et al., 2021). Non-invasive electrodes and alligator clips were placed on the rats before they were wrapped in a jacket. Then the rats were placed in a restraint tube. The rats were given time to wake up and given a 20-min acclimation period before the balloon was inflated with 2 mL of air. To mark the beginning and end of each CRD event, invasive ECG recordings were made simultaneously with non-invasive recordings as shown in Figure 1. The start of a CRD event was identified by a sharp increase in voltage generated by a single square pulse from a stimulator (GRASS s48 Stimulator). The stimulator was connected to the rat using a coax cable and alligator clamps, with one clamp positioned under a front limb and the other attached to the leg on the opposite side. The pulse from the stimulator not only marked the start of the CRD event but also initiated the inflation of the balloon. To ensure consistent force and timing, a 3D-printed syringe holder with a small motor was used. The motor was programmed to inflate the catheter balloon with 2 mL of air for 1 min. This process was repeated three times during the experiment, with 10-min intervals between each inflation. The CRD protocol was conducted on specific post-surgery days, which included days 5, 7, 9, 11, 14, 16, 19, and 21 (Kramer and Kinter, 2003; Krassioukov et al., 2009; Popok et al., 2016).

The entire experimental procedure was approved by the Purdue Animal Care and Use Committee (PACUC). The approval number associated with our project is 1810001814.

### 2.3. Data collection and analysis

During the non-invasive data collection, ECG electrodes were connected to a bio-amplifier on the Power Lab 26T system (AD Instruments, USA) at a sampling rate of 10 kHz. The recorded ECG signal underwent downsampling to 2 kHz and was then high-pass filtered using a cutoff frequency of 500 Hz, following the methodology described by Everett IV et al. in 2017. From the filtered signal, the integrated skin nerve activity (ISKNA) was obtained by integrating the absolute value over a 100 ms period using LabChart 8 software (AD Instruments, USA).

**Figure 2:**
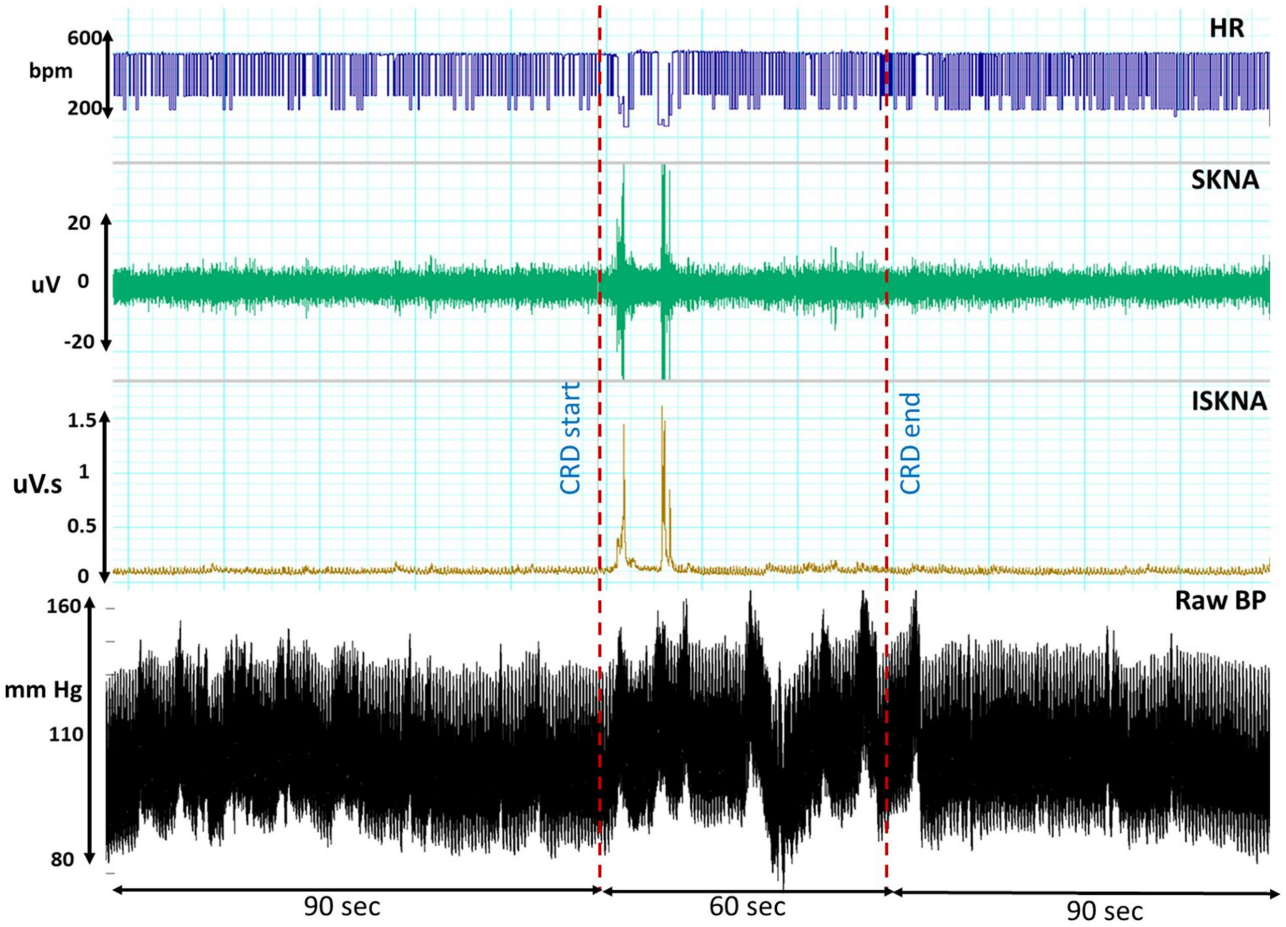
This illustrates the temporal profiles of raw blood pressure, heart rate (HR), skin nerve activity (SKNA), and integrated skin nerve activity (ISKNA) recorded during the colorectal distension (CRD) procedure that led to autonomic dysreflexia (AD).

The occurrence of the AD event was identified by observing the rise in blood pressure, specifically an increase of more than 15 mmHg, prior to the onset of bradycardia, which was visually confirmed. This distinct pattern, as illustrated in Figure 2, is characterized by an initial elevation in blood pressure followed by a subsequent decrease in heart rate (Suresh et al., 2022a).

To detect burst activity, a dual threshold algorithm was applied. This algorithm calculated the amplitude at each time point within the frequency range of 500 to 995 Hz. The computed amplitudes were normalized by the average amplitude of the entire series. Two thresholds, based on normalized amplitudes, were established: a higher threshold for burst onset detection and a lower threshold for burst termination. A burst was considered to occur when the signal’s amplitude crossed the higher threshold and lasted until the amplitude fell below the lower threshold. The analysis of SKNA and burst activity was performed using the “neurodsp” library in Python, as described by Cole et al. (2019). Parameters such as the number of bursts per second and burst duration were also calculated.

For HRV and SKNA features, normalization was conducted per rat per experiment day. Data from 1 min before, during, and after each AD event were exported into Origin Pro for further analysis. Peak detection was achieved using a local maximum method, with the baseline defined as the mean of 180-s windows. Peaks exceeding 20% of the threshold were considered significant. The time delay between the onset of AD and the increase in SKNA activity, root mean square of RMSSD, and HR were calculated.

Simultaneously, ECG recordings were obtained from implanted telemetry devices throughout each experiment day, with a duration of 50 min. This allowed data collection during both the baseline and adverse event periods. The Lomb periodogram within LabChart 8 software (AD Instruments Inc., Colorado Springs, CO) was utilized to calculate the power spectrum of frequencies present in the HRV, derived from the RR-interval variation. The Lomb periodogram, employing the method of least squares, facilitated the fitting of sine waves and the computation of power spectra at specified frequencies, following the methodology described by Li et al. (2019). Prior to the Lomb periodogram, the filtered ECG signal underwent windowing and linear detrending. Power spectrum plots were generated and categorized into three bands: VLF, LF, and HF. In the rat model, the VLF band ranged from 0 to 0.2 Hz, the LF band ranged from 0.2 to 0.75 Hz, and the HF band ranged from 0.75 to 2.5 Hz.

The integrated SKNA data were also analyzed in these frequency bands. Furthermore, in our study, we utilized the entire frequency band ranging from 0 to 10 Hz to comprehensively analyze the frequency components of interest. Specifically, we focused on frequencies beyond the conventional 2 Hz range to investigate HRV and gain insights into the autonomic nervous system’s influence on cardiac regulation. These frequency bands were selected to assess the impact of the autonomic nervous system on bodily functions, as established by Silva et al. (2017) and Zajączkowski et al. (2014).

Specific sections of data collected during the baseline and CRD events that led to AD were extracted and subjected to analysis. The dominant frequencies were detected using a 128,000-point fast Fourier transform size with 93.75% overlap, following the approach outlined by Liu et al. (2021).

## 3. Results

In this study, we employed min-max normalization to rescale the features of each day for every rat. This normalization method allows for fair comparison and combination of features across different days by transforming the data to a common scale. Data analysis was conducted per trial, enabling a detailed examination of the collected data for each specific day of the experiment. These meticulous steps were taken to ensure precise and reliable analysis while mitigating potential confounding factors.

### 3.1. Time delay analysis

**Figure 3:**
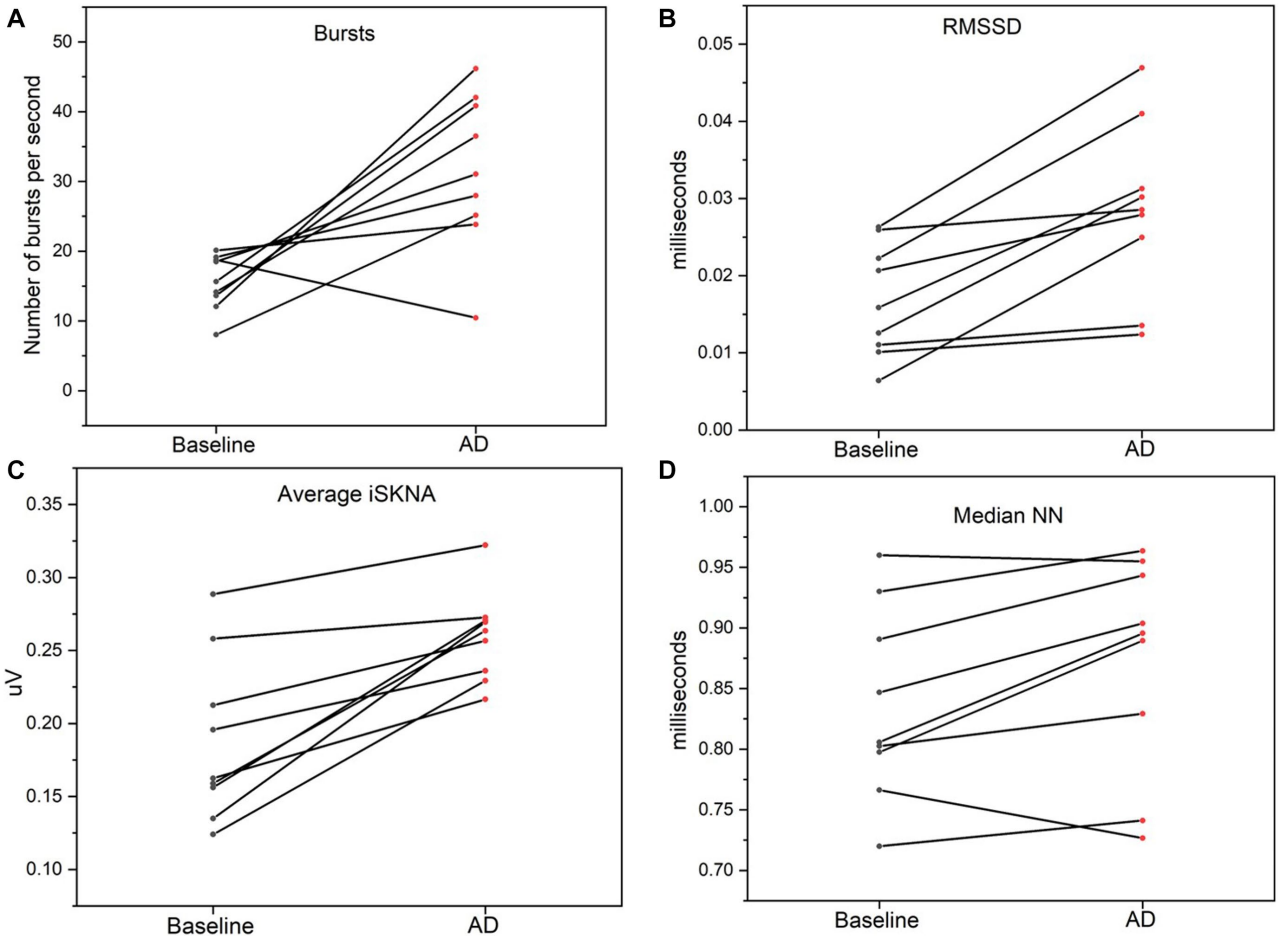
**(A)** Bursts per second was significantly higher during autonomic dysreflexia (AD) compared to baseline. **(B)** Average integrated skin nerve activity (ISKNA) during baseline was significantly increased during AD compared to baseline. **(C)** Root mean square of successive differences (RMSSD) was significantly higher during AD compared to baseline. **(D)** Median NN was significantly higher during AD compared to baseline.

Both HRV features, including medianNN and RMSSD, were significantly higher during AD compared to baseline. Similarly, average ISKNA and the number of bursts per second were significantly higher during AD as seen in Figure 2. The study found that SKNA bursts and ISKNA activity increased before SBP increased, indicating the onset of AD. The average time delay between the onset of CRD and the increase in ISKNA and SKNA bursts was 5.6 ± 4.5 s and 5.3 ± 5.2 s, respectively. An illustration showcasing this phenomenon can be found in Figure 3.

**Figure 4:**
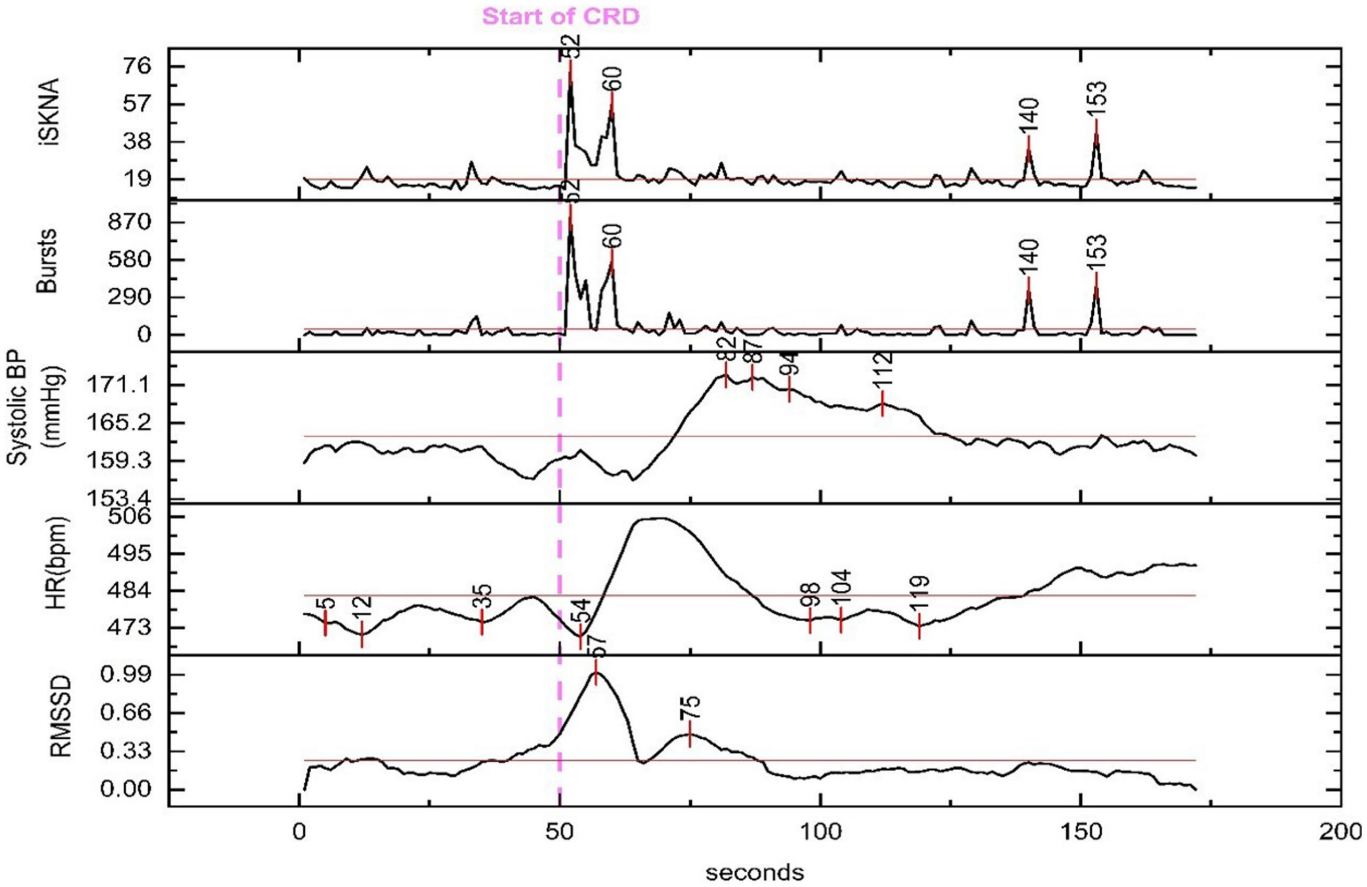
Physiological features extracted for time delay analysis from a representative animal. The dashed line marks the beginning of the colorectal distention (CRD) event that led to autonomic dysreflexia (AD) and the time for each peak is marked throughout the recording. An increase in skin nerve activity (SKNA) features and root mean square of successive differences (RMSSD) along with a decrease in heart rate (HR) is seen shortly after the onset of CRD.

The increase in SBP occurred, on average, 23.4 ± 18.0 s after the inflation of the balloon catheter, which inducted AD ≈ 70% of the time. Changes in RMSSD, an indicator of HRV, were observed at 9.4 ± 9.3 s after the onset of AD. The decrease in heart rate was detected at 35.8 ± 34.4 s after the beginning of the AD event. Additionally, the variation between the HR parameters is larger compared to the SKNA parameters (Figure 4).

The ANOVA (analysis of variance) revealed significant differences in the physiological parameters between the baseline and AD groups. Specifically, ISKNA (*p* = 0.04) and bursts per second (*p* = 0.01) exhibited distinct patterns, indicating substantial disparities between the baseline and AD states. Additionally, medianNN showed a significant difference (*p* < 0.001), highlighting contrasting characteristics between the baseline and AD groups. These findings provide valuable insights into the unique physiological responses associated with ISKNA, burst per second, and medianNN, underscoring the distinctive features of the baseline and AD states.

Furthermore, the Tukey Kramer Post-hoc Test indicated significant differences (*p* < 0.05) in the time delay from the start of CRD to the increase in various features, including ISKNA vs. SBP, bursts per second vs. SBP, RMSSD vs. SBP, bursts per second vs. HR, and ISKNA vs. HR. These results highlight the significant distinctions in the temporal relationships between ISKNA and the other variables.

### 3.2. Frequency domain analysis

#### 3.2.1. SKNA and ISKNA (0 to 10 Hz band)

**Figure 5:**
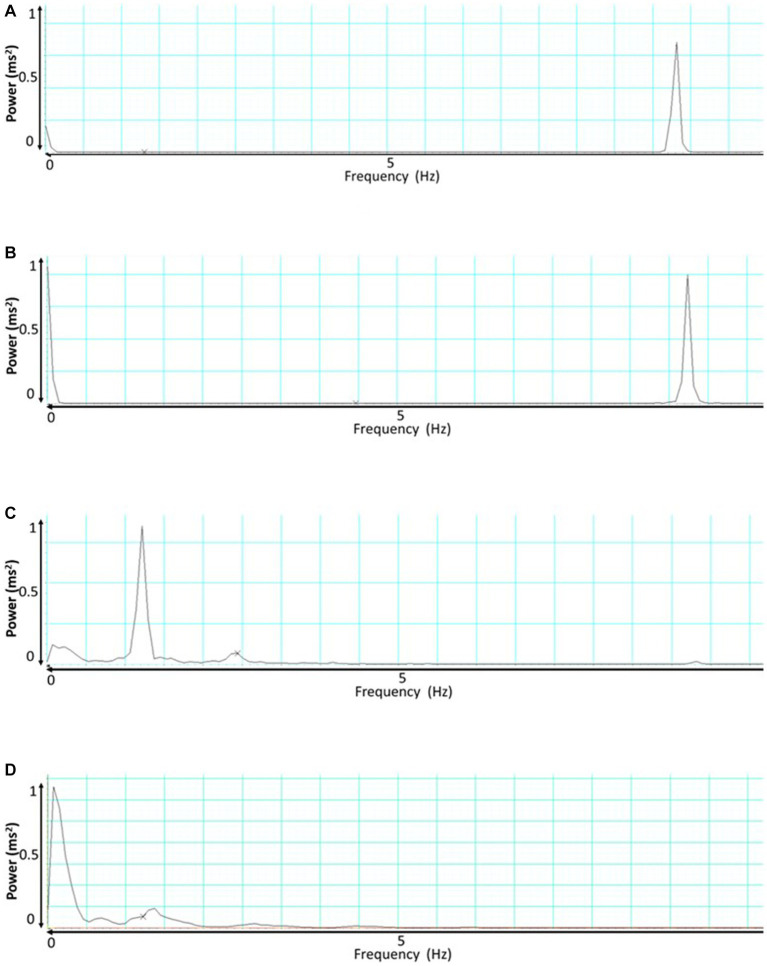
This presents the frequency analysis of skin nerve activity (SKNA) and integrated skin nerve activity (ISKNA) signals during baseline and autonomic dysreflexia (AD). **(A)** Shows the SKNA signal during baseline, while **(B)** displays the SKNA signal during AD. Similarly, **(C,D)** exhibit the ISKNA signal during baseline and AD, respectively.

During the baseline measurement, the SKNA signal exhibited a dominant frequency of approximately 8.23 ± 0.67 Hz. However, when an AD event occurred, there were distinct changes in the frequency profile. Two notable peaks emerged during this period: the first peak was observed at around 7.23 ± 0.78 Hz, and a dominant peak was also evident at 0.001 Hz as shown in Figures 5A,B.

Conversely, the baseline analysis of the ISKNA signal revealed a dominant frequency of approximately 1.4 ± 0.23 Hz. Interestingly, when an AD event took place, a significant shift in the dominant frequency occurred. The dominant frequency moved to a lower value, approximately 0.076 ± 0.12 Hz shown in Figures 5C,D.

#### 3.2.2. ISKNA (VLF, LF, and HF bands)

**Figure 6:**
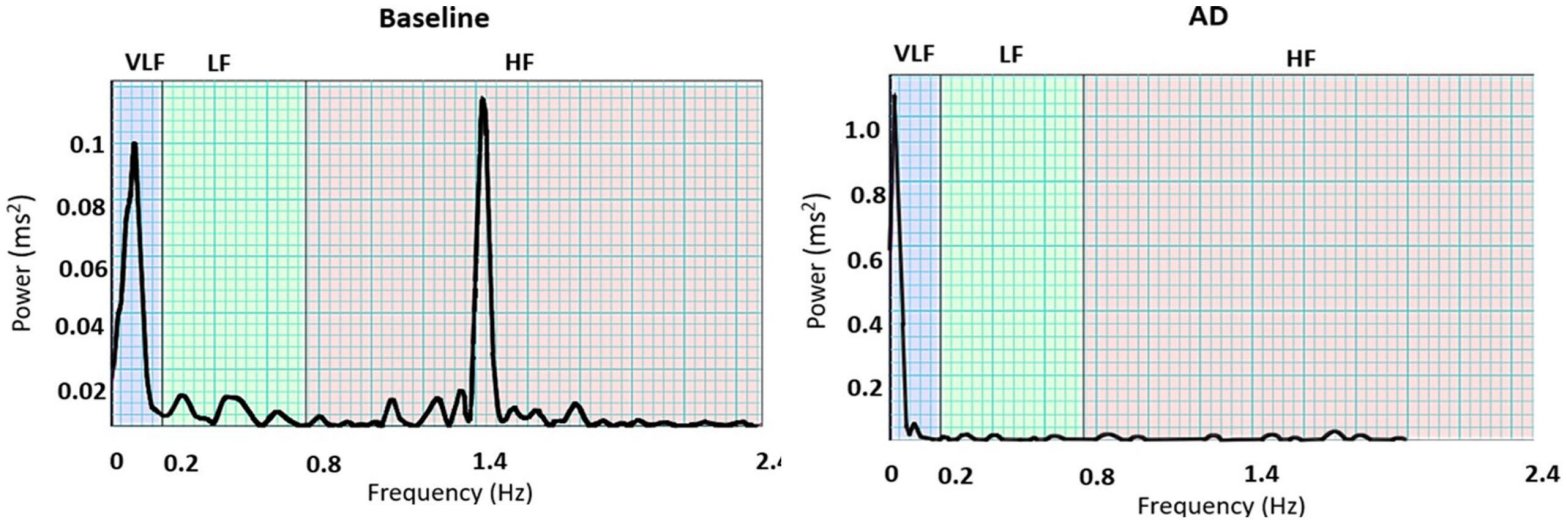
PSD plots during baseline and autonomic dysreflexia (AD) for one subject: Dominant frequencies during baseline were seen in both the very low frequency (VLF) and high frequency (HF) bands, while VLF is dominant during AD.

The dominant ISKNA frequency during AD was observed to be in the LF band. This was supported by a significant difference (*p* < 0.05) between the average dominant frequency during baseline (0.19 ± 0.11 Hz), typically associated with the HF band, and the average dominant frequency during AD (0.07 ± 0.01 Hz), indicating a shift towards the LF band. The PSD plots in Figure 6 depict the power spectral density of AD and baseline.

#### 3.2.3. Heart rate variability

Dominant frequencies of RR intervals were analyzed and normalized for all nine subjects. The average VLF power during baseline was 37.8%, while the average VLF power during AD was 44.2%. LF power was the lowest for both baseline (11.7%) and AD (12.0%). Average HF power was 39.0% during baseline and 24.3% during AD. The dominant frequencies for each subject are summarized in Table 1.

**Table 1:** Dominant frequencies for each subject.

	VLF (%)		LF (%)		HF (%)		LF/HF	
Sub	Baseline	AD	Baseline	AD	Baseline	AD	Baseline	AD
1	30.3	36.8	15.2	15.8	46.2	38.5	0.354	0.443
2	56.3	39.1	4.1	10.8	34.3	32.9	0.186	0.355
3	45.1	47.1	11.0	12.6	32.6	28.3	0.345	0.564
4	49.8	48.7	9.9	8.7	28.9	31.9	0.492	0.309
5	31.7	62.5	8.7	10.6	25.4	25.3	0.432	0.395
6	46.4	78.4	19.2	6.6	30.9	13.5	0.656	0.498
7	12.9	16.1	13.2	14.8	58.7	54.6	0.225	0.270
8	27.9	34.7	16.3	17.5	47.0	40.2	0.368	0.499
9	39.6	34.4	7.3	10.9	47.1	43.3	0.196	0.316
Aver-age	37.8	44.2	11.7	12.0	39.0	34.3	0.362	0.405
	(12.8–56.3)	(16.1–78.4)	(4.1–19.2)	(6.5–17.4)	(25.3–58.7)	(13.5–54.6)	(0.27–0.56)	(0.18–0.36)

**Figure 7:**
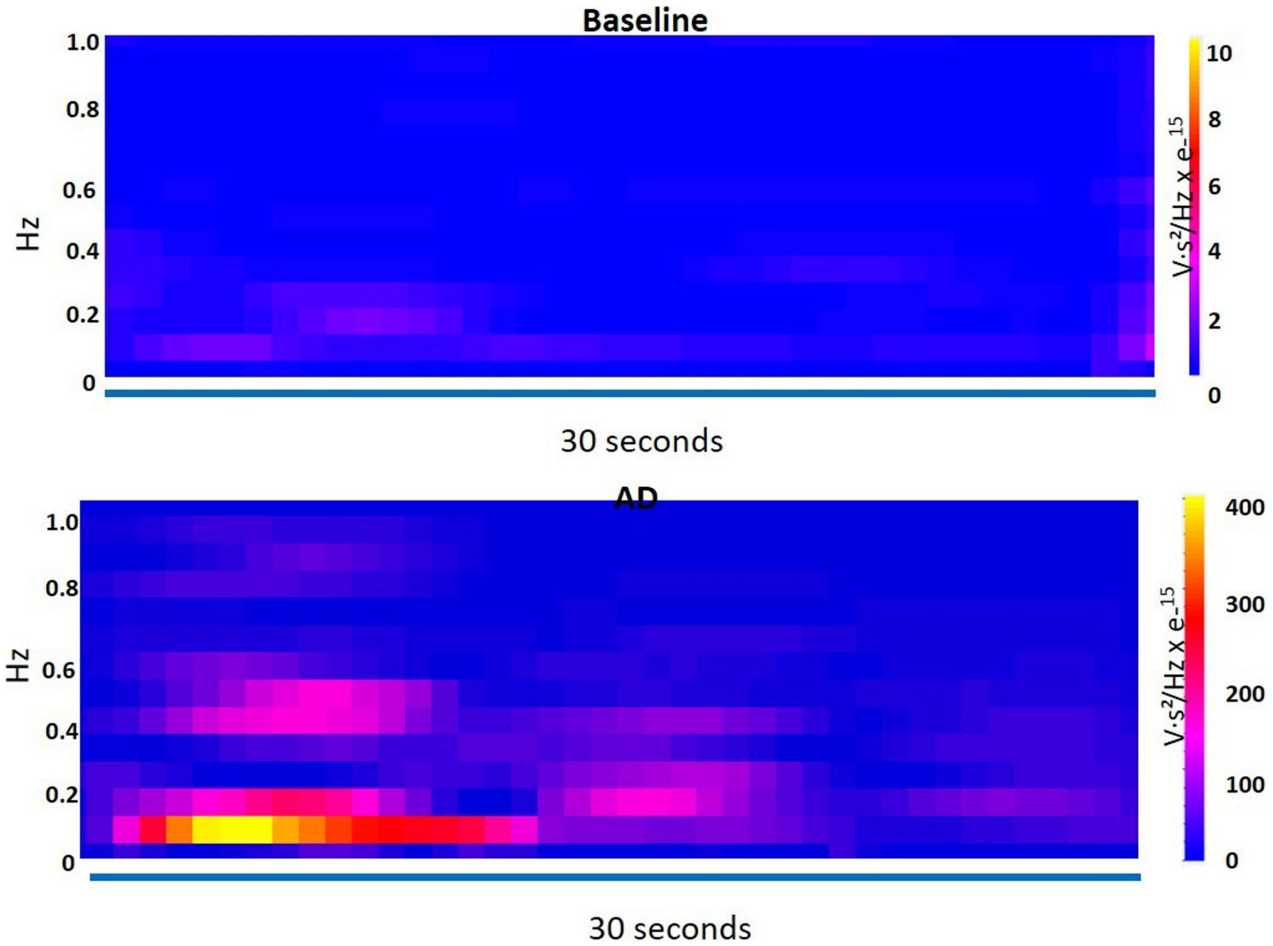
Time-frequency plot shows the dominance of very low frequency (VLF) during autonomic dysreflexia (AD) as compared to the baseline.

An ANOVA was performed to compare the measurements between the baseline and AD conditions for each frequency band and LF/HF ratio. The results revealed no significant difference between baseline and AD for VLF power (*p* = 0.38) and LF power (*p* = 0.13). However, there was a significant decrease in HF power (*p* = 0.017) during AD compared to baseline. Furthermore, the LF/HF ratio was significantly higher (*p* = 0.0328) during AD compared to the baseline. The time-frequency plot in Figure 7 displayed the dominance of VLF during AD in contrast to the baseline.

These findings indicate that while VLF and LF power did not differ significantly between the baseline and AD conditions, HF power was significantly lower and the LF/HF ratio was significantly higher during AD. The time-frequency plot further supports the observation of increased VLF dominance during AD.

Further MANOVA (Multivariate Analysis of Variance) analyses were performed to explore these differences in more detail for the time domain (ISKNA, bursts per second, RMSSD, medianNN) and frequency domain (VLF, LF, HF, LF/HF) features separately. The time domain features showed a highly significant distinction (*p*-value = 0.0002), indicating a clear dissimilarity between the AD and baseline groups in this domain. Similarly, the frequency domain features exhibited an even more pronounced significance (*p*-value = 1.07634e-07), indicating a substantial and statistically significant differentiation between the AD and baseline groups in this specific domain. These findings provide robust evidence of significant variations between the AD and baseline groups across both the time domain and frequency domain features, as evidenced by the separate MANOVA analyses.

## 4. Discussion

SKNA features were detected noninvasively and before the typical rise in SBP as measured through telemetry implants during AD events. This study focused on further understanding changes in sympathetic and parasympathetic activity in response to AD in both time and frequency domains. Using implanted telemetry ECG, we compared the timing of ISKNA and nerve bursts to the resultant increase of SBP during AD. We anticipated that activation of the stellate ganglion, as measured through SKNA, would precede the increase of SBP through baroreflexes. For newly spinal cord injured individuals it takes time to learn their telltale symptoms of AD and then determine what the source or trigger of this episode may be, such as a lack of voiding of the urinary bladder, noxious stimuli not felt below the injury, or bowel impaction. Thus, there may be medical benefits of such an AD detection system.

### 4.1. Time domain analysis

AD is characterized by a large increase in sympathetic activity that leads to an increase in blood pressure. The sympathetic activity was detected through a significant increase in SKNA features, including average ISKNA and the number of bursts per second during AD. The parasympathetic activity was also detected during AD through the increase in median NN and RMSSD.

The gold standard of AD detection is an increase in SBP, but this study exposed activation of the sympathetic nervous system was first detected through SKNA. Burst activity was detected using a noninvasive ECG recording, which encourages the development of wearable solutions for persons with SCI that suffer from AD. Therefore, burst activity and ISKNA has the potential to detect AD prior to the symptomatic manifestation of paroxysmal hypertension that is currently used by persons with SCI to detect the occurrence of an AD event. Throughout the study, the bursts per second count, which reflects the burst activity of the SKNA signal, exhibited distinct patterns. On day 5, the bursts per second count was recorded at 84.76 bursts/s, indicating a high level of burst activity at the onset of AD. It is important to note that on this particular day, there were only a small number of reported AD events, which was less than half of the count observed on other experimental days. This suggests that the burst detection algorithm may have captured low-amplitude spikes as bursts during the recovery period.

From day 7 to day 16, the burst per second count gradually decreased, reaching an average value of 20.19 bursts/s on day 16. This decline in burst activity indicates a reduction in the occurrence of AD-related bursts during this period. However, starting from day 19, there was a notable increase in the burst per second count. On day 19, the average burst per second count rose to 37.57 bursts/s, and on day 21, it further increased to 41.35 bursts/s. This upward trend in burst activity suggests a resurgence of AD-related bursts in the later stages of the study.

The larger variation within HR parameters showed that parasympathetic activity did not activate at a uniform rate after the onset of AD. Instead, parasympathetic activity occurs in response to the increase of systolic blood pressure and is likely activated through changes in baroreceptors in the cardiovascular system. Therefore, using SKNA activity to detect the onset of AD would be more accurate. Providing early detection of AD before symptoms arise would provide persons with SCI additional time to identify and eliminate AD triggers to prevent the onset of major symptoms and outcomes associated with AD. Although the onset of SKNA spikes averaged only 18 s prior to the anticipated increase in SBP in this rat study, this may have clinical value for paralyzed persons.

### 4.2. Frequency domain analysis

The ISKNA frequency domain analysis revealed LF dominance during AD and HF dominance during baseline, which follows the literature, which has found LF to be dominant during disease states (Meng et al., 2022). The use of SKNA has been shown to be predictive of increased cardiac sympathetic tone, such as heart failure, resulting in a significant decrease in LF components. SKNA was shown to be more accurate compared to HRV in assessing cardiac sympathetic activity in canines with myocardial infarction (Zajączkowski et al., 2014). Because cardiac and skin sympathetic innervations are linked, sympathetic nerves from both structures activate at the same time. HF dominance during baseline indicated parasympathetic activity as confirmed through HRV frequency domain analysis. LF dominance during AD clearly exhibited sympathetic hyperactivity using ISKNA analysis, which was not defined during power spectral HRV analysis due to loss of sympathetic nervous tone in patients with SCI.

In our frequency analysis of the SKNA signal within the 0 to 10 Hz range, we observed a distinct frequency component around 8 Hz. Notably, during the occurrence of AD, we observed a subtle shift in this frequency component, moving towards approximately 7 Hz. This frequency shift implies the presence of bradycardia, a reduction in heart rate, during AD episodes. These findings highlight the potential association between changes in HRV and cardiovascular responses during AD.

Our HRV frequency domain analysis detected parasympathetic activity during baseline with a significant increase in HF, and a difference in LF/HF ratio that revealed a shift of sympathovagal balance toward sympathetic dominance during AD. There was no significant difference in VLF between baseline and AD, which could be because of the SCI, which disconnects the communication of the sympathetic nervous system between the lungs and the heart. LF power also was not significantly different between baseline and AD, which could be explained by the sympathetic and parasympathetic activity fluctuations during AD. Our data suggest HRV frequency domain analysis, which emphasized the detection of parasympathetic parameters, may not be optimal for AD studies due to the lack of differences in LF and VLF components. In contrast, subcutaneous nerve activity has been shown to be more effective in estimating cardiac sympathetic tone.

## 5. Conclusion

We determined that certain SKNA parameters can be detected noninvasively before the gold standard increase in blood pressure. The frequency domain analysis of ISKNA has been found to be a useful technique in the identification of biomarkers for detecting AD in rats, with a specific emphasis on the LF band during the progression of the disease. These biomarkers can aid in the development of a universally applicable approach for the early detection and monitoring of AD, which may have significant clinical implications. SKNA frequency domain features can noninvasively detect the early onset of AD in paralyzed humans. Such a system would advantageously alert SCI individuals of impending AD events prior to severe symptoms, preventing long-term cardiovascular complications. Identifying these parameters provides insights into autonomic function during AD events, which can inform targeted interventions for AD patients with SCI.

## Data availability statement

The datasets used and/or analyzed during the current study available from the corresponding author upon reasonable request.

## Ethics statement

The animal studies were approved by Purdue Animal Care and Use Committee (PACUC). The studies were conducted in accordance with the local legislation and institutional requirements. Written informed consent was obtained from the owners for the participation of their animals in this study.

## Author contributions

AK designed and carried out the experiments, analysis, and drafted the manuscript. SP provided guidance with data analysis and edited the manuscript. ZA and CC provided invaluable support while conducting experiments. THE provided guidance using skin nerve activity physiological recording. BSD conceived, coordinated, and facilitated this research.

## Funding

We would like to thank the Indiana State Department of Health through the Indiana Spinal Cord and Brain Injury Research Fund (PI: BSD) and the Department of Defense Congressionally Directed Medical Research Programs Spinal Cord Injury Research Program (SC190164 W81XWH-20-1-0725) for supporting this research. We would like to express our gratitude to the National Institutes of Health (NIH) for their support of our research through the NIH R01HL158952 grant (PI: Everett).

## Conflict of interest

The authors declare that the research was conducted in the absence of any commercial or financial relationships that could be construed as a potential conflict of interest.

## Publisher’s note

All claims expressed in this article are solely those of the authors and do not necessarily represent those of their affiliated organizations, or those of the publisher, the editors and the reviewers. Any product that may be evaluated in this article, or claim that may be made by its manufacturer, is not guaranteed or endorsed by the publisher.
